# Histomonosis - an existing problem in chicken flocks in Poland

**DOI:** 10.1007/s11259-015-9637-2

**Published:** 2015-05-15

**Authors:** Beata Dolka, Artur Żbikowski, Izabella Dolka, Piotr Szeleszczuk

**Affiliations:** Department of Pathology and Veterinary Diagnostics, Faculty of Veterinary Medicine, Warsaw University of Life Sciences-SGGW, Nowoursynowska 159c St., 02-776 Warsaw, Poland

**Keywords:** Histomonosis, Chickens, Broiler breeders, Re-emerging disease

## Abstract

Histomonosis (histomoniasis, blackhead), beside coccidiosis, belongs to the most important parasitic protozoan diseases in poultry. So far *Histomonas meleagridis* infections with varied mortality rates have been mainly diagnosed in young turkeys. Recently an increasing number of cases have been reported in chicken flocks in Europe resulting in economic losses. It is thought that this situation is predominantly caused by a complete withdrawal of the effective antihistomonals in the EU. Authors listed the selected outbreaks of histomonosis in 10 chicken flocks originated from different farms of 4 regions in Poland: 8 broiler breeder flocks (at mean age of 33 weeks) and 2 commercial layers flocks (at mean age of 38 weeks). This study reported here naturally occurring case of *H.meleagridis* infection in commercial broiler breeder (BB) flock line ROSS 308 at the age of 16 weeks. We showed acute form of infection with characteristic necrotic foci in the liver, and ulcerative typhilitis. Beside the liver and caeca, the multiple histomonads, lymphoid tissue depletion and heavy destruction in the bursa of Fabricius were observed. Additionally, the absence of systemic diffuse histomonads and lack of *Heterakis gallinarum*, caecal worm eggs in faecal samples were noted. PCR technique enabled to detect the presence of *H.meleagridis* genetic material in the investigated tissue samples. Authors indicate that histomonosis can be considered as re-emerging infectious diseases in chicken flocks of intensive production system.

## Introduction

Histomonosis (also known as Blackhead) is one of the most important diseases in poultry caused by the flagellated protozoon *Histomonas meleagridis*. The diseases is mainly associated with the turkeys, due to their susceptibility to *H.meleagridis* and economic importance, however other bird species, such as: chickens, pheasant, peafowl, quail, partridge, guinea fowl, duck and ostrich may also be prone to infection. Although the first cases were described in 1895 in turkeys and in 1900 in chickens, the disease still poses a serious threat to the poultry industry due to the mortality (McDougald 2005; Hess and McDougald 2013). For many years chemotherapeutics have been used in histomonosis prevention and treatment, especially nitroimidazoles (metronidazole, dimetridazole, ronidazole), nitrofurans (furazolidone), arsenical derivatives (e.g., nitarsone, roxarsone, acetarsol). However, their use in poultry has been banned in countries belonging to the European Union (EU), due to the serious risk to public health (possible toxic and cancerogenic effects). The ban of these products in combination with the changes in animal husbandry were followed by an upsurge in reported cases in poultry (CEC 2003; Hess et al. 2006; Callait-Cardinal et al. 2007; EFSA 2013; Hess et al. 2015). As a consequence, the problem of histomonosis has escalated in chickens. Histomonosis in chickens has been noted in some European countries i.a. Belgium (layers, Esquenet et al. 2003), Austria (layers, Grafl et al. 2011), in the Netherlands (layers, Van der Heijden and Landman 2011), in Denmark (layers, Stokholm et al. 2010), Germany (broilers, Popp et al. 2011; layers, Hafez et al. 2001; chickens, Hauck et al. 2010), also in the USA (leghorn pullets, Homer and Butcher 1991) and in Asia: Malaysia (broilers; Ganapathy et al. 2000), India (broiler breeder flock; Banerjee et al. 2006), (broilers; Patra et al. 2013). Moreover, the serological evidences for *H.meleagridis* distribution in layer chicken flocks kept in different housing systems (Grafl et al. 2011; Van der Heijden and Landman 2011) and genotypic variations of parasite isolates have been shown (Bilic et al. 2014). It is considered that the type of poultry farming and hygienic conditions significantly influence the spread of the disease (Esquenet et al. 2003; Grafl et al. 2011). Renewed efforts in histomonosis investigations have led to new insights into the epidemiology and molecular characterization of *H.meleagridis* (Grabensteiner et al. 2006; Hess et al. 2006; Hu et al. 2006; Bilic et al. 2014; Lotfi et al. 2014; Hess et al. 2015). In order to improve control strategies, the research studies have boosted to develop of new treatment and prophylactic strategies including vaccination (Hu and McDougald 2002; Bleyen et al. 2009; Hafez et al. 2010; Van der Heijden et al. 2011; Liebhart et al. 2013; Hess et al. 2015).

The main goal of current study was to investigate the prevalence of natural infection of *Histomonas meleagridis* in commercial broiler breeder flock as well as highlight the problem of histomonosis involving chickens raised in the intensive farming systems.

## Materials and methods

### Case presentation, clinical history, treatment

Six 16-weeks-old chickens from affected broiler breeder flock line ROSS 308 which showed clinical signs before death were submitted to the Division of Avian Diseases, Faculty of Veterinary Medicine at the Warsaw University of Life Sciences. Case history revealed that the chicken farm was situated about 100 m from a road which was used to transport feed, chickens and poultry manure; 1 km from other broiler breeder farm, and about 2 km from a commercial turkey farm. In the investigated farm the parent stock was kept for the third time with the initial chickens number of 30 081. The farm was operated according to the all-in all-out system. There was 2 weeks interval between each introduction of new chickens to the production house, during which the hot cleaning and disinfection were performed with the use of Rapicid solution (Evans Vanodine International, UK), liquid ammonia, sodium hypochlorite solution, Aldekol Des® 03 (Ewabo, Germany), formaldehyde fumigation (used twice), and thermal disinfection (open flame). The flocks had no access to outdoor areas. The breeding conditions, vaccination program and feeding were conducted in accordance with the standards for this type of poultry production system. Chickens were vaccinated (at 3 days of age) against coccidiosis and they were routinely dewormed at 8 and 14 weeks of age through the administration levamisole in drinking water. At the beginning of rearing period the birds were kept in three grow-out houses (two facilities held 24 000 hens, one 6 000 roosters) and one house remained empty. In the 5th week of rearing the hens were divided into two flocks by moving 15 819 birds into the empty production house. In this house, the onset of disease was noted in 16-weeks-old chickens. Serum samples were randomly collected from 23 chickens at the age of 16 weeks and tested using ELISA (Idexx, USA) for antibodies against infectious bursal disease virus (IBDV) and infectious anemia virus (CIAV). Before the onset of disease, the flock was vaccinated against IBD according to the immunoprophylaxis program. Vaccination against CIA was planned at 19 weeks of age. Serological investigation showed 100 and 95.5 % positive samples for antibodies against IBDV (geometric mean titer 6371) and CIAV (S/N 0.081) respectively.

The owner of the investigated poultry farm was advised to treat the infected flock with herbal preparation composed of natural extracts from plants and aromatic substances (Fitotril, Chemifarma, Italy) in a dose of 200 mL/100 L of water for 7 days. Additionally, flubendazol (Solubenol, Janssen Animal Health, Belgium) was used for deworming the chickens at 19 weeks of age in a dose of 1.43 mg/kg body weight daily, administered orally in drinking water for 7 days. Moreover, the disinfectants used for foot mats in front of chicken house were replaced with the solution contained glutaraldehyde. The litter used to cover the losses was replaced with a new one free from soil and earthworms.

### Necropsy and histopathological examination

During necropsy, tissue samples (liver, spleen, small intestines, caeca, kidney, bursa of Fabricius) were collected for histopathological examination. After fixation in 10 % buffered formalin, tissue specimens were processed, embedded in paraffin and finally stained with hematoxylin and eosin (H-E).

### Parasitological examination

The faecal samples were collected for examination by using normal saline solution direct smear wet preparation technique. The liver and caeca samples were stained with Hemacolor® kit (Merck, Germany) and observed under microscope (Olympus, Japan). The visual inspection for intestinal parasites, including *Heterakis gallinarum* in the caeca was undertaken.

### Microbiological examination

Microbiological examination were performed on samples from liver, spleen and caeca, which were cultured in standard and selective media (Columbia agar with 5 % sheep blood, MacConkey agar).

### Polymerase chain reaction (PCR)

PCR technique was used to detect the presence of *Histomonas meleagridis* genetic material in the liver and intestinal samples. A 25 mg of organ material was taken for DNA extraction using Chelex 100 (Biorad, Poland) and Sherlock AX kit (A&A Biotechnology, Poland) according to manufacturers’ instructions. Amplification of *H.meleagridis* DNA was done using a species specific pair of primers Hmf 5′-GAAAGCATCTATCAAGTGGAA-3′, Hmr 5′ GATCTTTTCAAATTAGCTTTAAA-3′ previously described by Grabensteiner and Hess (2006). The each reaction mixture consisted of 25 μl DreamTaq PCR Master Mix (2X), 0.5 μM of each primer, 4 μl DNA and PCR-clean water (added up to a volume of 50 μl). PCR conditions were used in accordance with Grabensteiner and Hess (2006): initial denaturation at 95 °C for 5 min, followed by 35 cycles: denaturation at 95 °C for 60 s, annealing at 50 °C for 45 s, extension at 72 °C for 120 s. Thereafter, the samples were maintained at 72 °C for 10 min for final extension step. Amplification products (10 μl) were analyzed by agarose gel (1.2 %) electrophoresis after ethidium bromide staining and visualized under UV light (UVP, USA). Fragment sizes were determined with reference to a 100 bp ladder (Thermo Fisher Scientific Inc., USA). The expected PCR product size was 574 bp.

## Results

### Clinical signs and mortality

For the first 15 weeks of rearing period, the chickens showed no obvious clinical signs, even though mortality was recorded (1.2 %). In details, between 1 and 4 week the mortality was 1.33 % (99.8 birds/per week), then 1.05 % between 5 and 15 week (15.1 birds/per week, after moving to the new house). Beginning from 16 weeks of age, the symptoms were noticed. The chickens showed untypical behaviour, manifested by aversion to moving, sitting on hocks, depression, ruffled feathers, drooping wings. Additionally, decreased of feed and water uptake were noted. The mortality reached 5.01 % (49.6 birds/per day; 347.2 birds/per week) within approx. 2 weeks with a peak on day 114 (102 birds/per day). After the peak the fluctuations in daily losses were observed (gradual reduction to 63 birds at 116 day, then increase up to 82 birds at 118 day). In total, 6.6 % (74.1 birds/per week) of chickens have died in this house between 5 and 19 weeks of age.

In Table 1 we presented the list of 10 selected outbreaks of histomonosis in commercial chicken flocks: 8 broiler breeder (BB) and 2 commercial layers flocks (CL). All flocks originated from different farms located on 4 of 16 voivodeships in Poland. Total of 4 voivodeships were located nearby and represented areas with intensive chicken (Mazovian and Greater Poland voivodeship) and turkey production in Poland (Warmian-Masurian, Lubusz and Greater Poland voivodeship). The histomonosis was diagnosed based on clinical data, pathological lesions (including histopathological examination in cases from three flocks) and PCR (in cases from five flocks). The mean age at onset of clinical signs was 33 weeks in BB and 38 weeks in CL flocks. The age of each flock was showed in Table 1.

**Table 1:** The list of selected outbreaks of histomonosis in commercial chicken flocks in Poland

Case	Date of diagnosis (month, year)	Type of production	Age of birds (weeks) at which clinical signs were noticed	Farm location (voivodeship)	Clinical & postmortem diagnosis (Yes/No)	Confirmation by PCR (Yes/No)	Undertaken therapy (Yes/No)
1	11.2002	BB	40	Mazovian	Yes	No	No
2	01.2010	BB	16	Mazovian	Yes	Yes	Nd
3	01.2010	BB	16	Lubusz	Yes	Yes	Yes
4	02.2010	BB	44	Mazovian	Yes	No	Nd
5	12.2010	BB	44	Warmian-Masurian	Yes	No	Yes
6	03.2011	BB	40	Mazovian	Yes	No	Yes
7	03.2011	CL	25	Greater Poland	Yes	Yes	Nd
8	03.2011	CL	51	Mazovian	Yes	Yes	Yes
9	08.2012	BB	19	Warmian-Masurian	Yes	No	Yes
10	02.2014	BB	42	Mazovian	Yes	Yes	Nd

*BB* Broiler Breeders, *CL* Commercial Layers, *Nd* no data

### Necropsy and histopathological examination

**Fig. 1:**
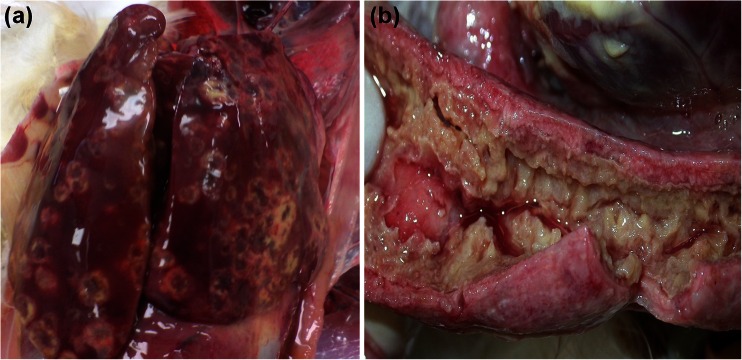
Macroscopic lesions in liver (**a**) and in caeca (**b**) in chickens

The external inspection of carcasses revealed severe emaciation, mat feathers and cloaca covered with faeces. During necropsy, an advanced liver damage manifested with enlargement, congestion with numerous yellow necrotic foci were noted (Fig. 1a). Additionally, we noted spleen congestion with beige rounded areas of necrosis, pulmonary congestion and obliterated structure of folds bursa of Fabricius. The kidneys were pale brown with increased urates in the ureters. We noted haemorrhagic inflammation of small intestine, thickening of the caecal wall with caseous inflammation and the ulceration of the mucosa (Fig. 1b).

**Fig. 2:**
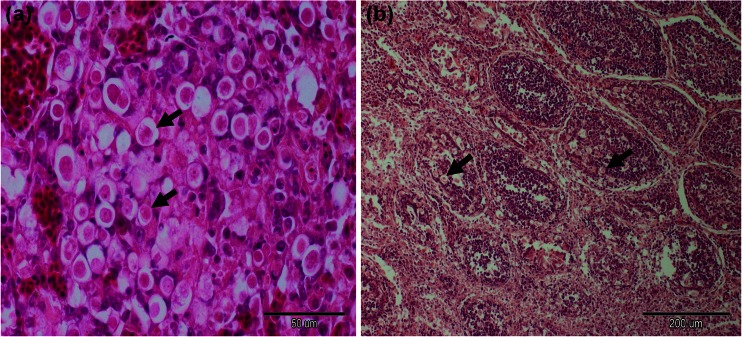
Histopathology of liver (**a**) and bursa of Fabricius (**b**) with multiple oval-shaped histomonads (*arrows*). Original magnifications: 400x (**a**), 100x (**b**); hematoxylin-eosin staining (H-E)

Histopathological examination showed massive multifocal necrosis and haemorrhages in the liver, moderate infiltration of mononuclear cells and multiple flagellated forms of the trophozoite dispersed within the organ (Fig. 2a). Furthermore, in the caeca were found trophozoites under the layer of exudation and multiple bacteria. Haemorrhage lesions were noted in kidneys, together with parenchyma degeneration and necrosis. Bursa of Fabricius showed depletion of lymphoid tissue and focal localization of trophozoites in the cytoplasm of phagocytes (Fig. 2b).

### Parasitological examination

The parasitological examination showed no presence of oocysts, nematodes or their eggs in faecal samples. Direct microscopic examination of the caecal contents as well as stained preparations did not reveal the presence of protozoa.

### Microbiological examination

Microbiological cultures enabled to identify numerous bacteria of *Gallibacterium* spp.

### PCR

**Fig. 3:**
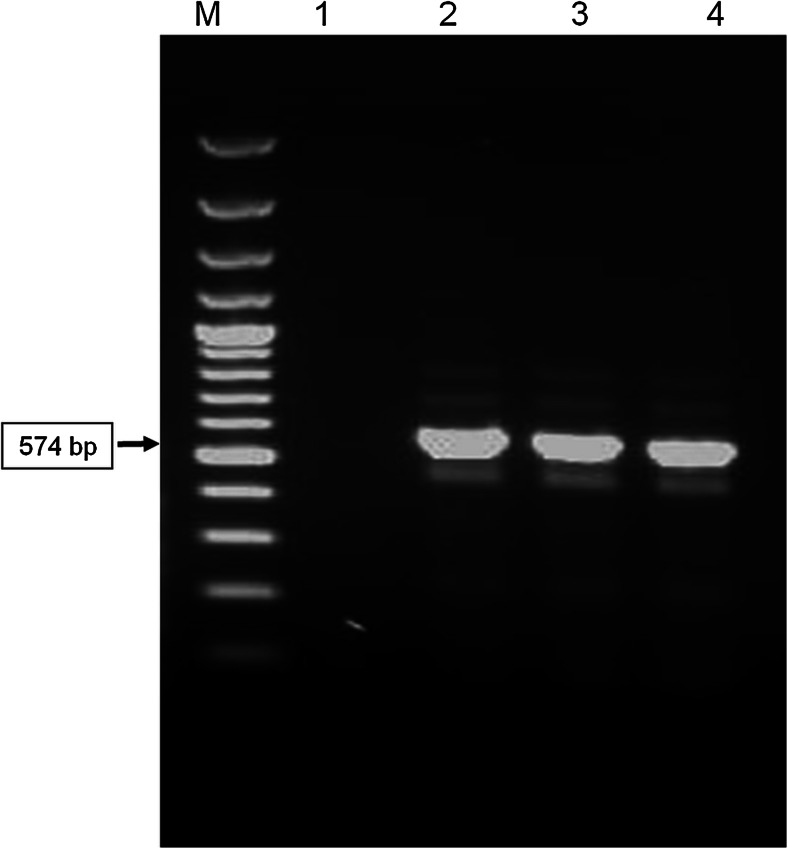
Agarose gel electrophoresis showing PCR amplification of fragment DNA *Histomonas meleagridis*. From left: M-molecular size marker (100 bp ladder); 1-negative control, 2-positive control; 3-liver sample; 4-caeca sample. The length of PCR product was 574 bp.

The parasite *H.meleagridis* DNA was detected in analyzed liver and caecal samples (Fig. 3).

## Discussion

Although several previous studies have investigated the prevalence of *H.meleagridis* infections in chickens (Tyzzer 1934; Gerth et al. 1985; Homer and Butcher 1991; McDougald 2005), the recent reports placed these birds next to the turkeys as the main affected poultry species (Callait-Cardinal et al. 2007; EFSA 2013; Hess and McDougald 2013; Hess et al. 2015). Many outbreaks of histomonosis in chickens have been reported in free-range systems, including organic flocks (Hafez et al. 2001; Esquenet et al. 2003; Stokholm et al. 2010; Popp et al. 2011). The present study supports the findings that chicken flocks in intensive production systems may be seriously affected. In contrast to the turkeys, histomonosis in chickens is known as usually less fatal, may be unnoticed and cause decrease in the performance indicators (McDougald 2005; Hess and McDougald 2013). Other reports documented more severe clinical signs, characterized by decrease in egg production and increased mortality in chicken flocks (Ganapathy et al. 2000; Esquenet et al. 2003; Popp et al. 2011). In comparison to above reports, experimental studies indicated on the role of chickens as an asymptomatic parasite carriers (Hess et al. 2006). Clinically, in our study the affected chickens exhibited mortality with apparent clinical signs. The observed symptoms were nonspecific, generalized and similar to those described in the literature (Popp et al. 2011; Hess and McDougald 2013). However, chickens showed no cyanosis of the head opposited to other reports (Ganapathy et al. 2000; Esquenet et al. 2003) and did not suffer from foamy or yellow diarrhoea, which is more typical for infected turkeys (Ganapathy et al. 2000; Cortes et al. 2004; McDougald 2005). In our study mortality rate was lower than recorded in chickens (Ganapathy et al. 2000; McDougald 2005; Popp et al. 2011) also after experimental infection (Desowitz 1951). On the other hand the noted mortality was higher than in pullets (Homer and Butcher 1991) or in commercial broilers (Cortes et al. 2004). In the examined flock, between 5 and 15 weeks, when no signs were observed the mortality was 1.05 % (0.1 % birds died/per week). After that, mortality in the flock reached 5.01 % in the period of disease spreading (2.2 % birds died/per week). Our results were similar to data obtained by Esquenet et al. (2003). However, according to the above authors, at time when no problems were observed mortality in the flock was approximately 0.20 % per week, then gradually rose to reach a peak of 1.05 %. Based on the field reports, mortality tends to be moderate (10–40 birds/week), but lingers for several weeks (McDougald 2005). Similarly to above author, chicken deaths were recorded constantly for many weeks. On the other hand, in our study, a rapid increase in mortality with the highest losses (347.2 birds/per week) was observed only during approx. 2 weeks of the disease (at 16 and 17 week).

Interestingly, we noted that older chickens were more likely to be affected by acute histomonosis than reported in previous literature (Desowitz 1951; Homer and Butcher 1991; Ganapathy et al. 2000; Popp et al. 2011). Zahoor et al. (2011) showed in experimental studies, that chickens may show frequently milder course of infection without clinical signs and mortality, despite of significant lesions in caeca. It is connected with the mechanism of early immune response activated in the intestinal mucosa, especially in caecal tonsils (Powell et al. 2009; Windisch and Hess 2010). Lotfi et al. (2014) suggested that, the genetic background of the chickens influences the reaction to infection with *H. meleagridis*.

It has been shown that hot and humid weather may exacerbated the histomonosis severity in chickens (Ganapathy et al. 2000). The field outbreaks in turkeys have occurred more frequently in the hottest months (Callait-Cardinal et al. 2007). Although in the present study, the outbreak of diseases occurred in winter month, the environmental conditions including microclimatic might be involved on manifestation of the disease.

According to the previous reports, the most prominent histomonosis-associated gross lesions were localised in the liver and caeca of chickens (Stokholm et al. 2010; Patra et al. 2013). The presence of multiple diffuse necrotic foci in the whole liver indicated on advanced and massive damage process. Our results were opposed by the general opinion, that rounded necrotic liver lesions are more typical for turkeys and often absent in chickens (Homer and Butcher 1991; Esquenet et al. 2003). The post mortem lesions in caeca, spleen and kidneys were characteristic for the disease (Homer and Butcher 1991; Ganapathy et al. 2000; Esquenet et al. 2003; Popp et al. 2011; Hess and McDougald 2013). Powell et al. (2009) found, that formation of pathological lesions in the course of histomonosis was less severe in chickens than turkeys, due to limited migration of protozoa from intestines to the liver. According to these authors, at 6–8 days post infection in chickens, regeneration of damaged caecal mucosa takes place and within a month the birds may recover, gain immunity, however they remain the transmitters of the parasite. We demonstrated multiple histomonads and destruction of the bursa of Fabricius in chickens. No histomonads were detected in spleen, kidneys, lungs. Although the spread of *H.meleagridis* to bursa was observed in chickens during a field outbreaks (Marx 1973; Cortes et al. 2004), the data obtained after experimental infections of chickens were not ambiguous (Grabensteiner et al. 2006; Zahoor et al. 2011). Many aspects of the pathogenesis of *H.meleagridis* infection have not yet been fully clarified and further studies are needed to investigate the parasite impact on the bursa of Fabricius in chickens.

Despite of histopathological lesions typical for histomonosis and detection DNA *H.meleagridis*, direct microscopic parasitological examination did not confirm the presence of protozoan parasites. Probably this could have been caused by a relatively long period that had passed after the death of birds, as identification of protozoa requires fresh material. Similarly to our results, other authors reported outbreaks of histomonosis in chickens without the presence of the gastrointestinal worms (Ganapathy et al. 2000; Cortes et al. 2004).

Histomonosis in chickens may be more severe by the co-infections which contribute significantly to the mortality (Stokholm et al. 2010; Popp et al. 2011). The degree of intestinal lesions depends on *Histomonas* virulence and factors damaging the intestinal mucosa, i.e., coccidia (*Eimeria tenella)*, nematode, bacteria (*E.coli, Clostridium perfringens*) and mycotoxicoses (Ganapathy et al. 2000; McDougald and Hu 2001; McDougald 2005). Authors suggested that *Gallibacterium* spp. might be involved in the disease picture. Previous studies showed that chickens may serve as the preferable host for those bacteria. *Gallibacterium* spp*.* may constitute a part of their normal flora of the respiratory and genital tracts. However, *Gallibacterium* isolates have also been recovered from various pathological lesions in chickens (Bojesen et al. 2003; Stokholm et al. 2010). Bojesen et al. (2003) reported higher prevalence of *Gallibacterium* spp. in chickens from production systems with moderate or low levels of biosecurity. Although the source of infection in the presented case was not known, the possible deterioration in hygienic conditions could not be excluded. Moreover, the introduction of CIAV to the flock, may indicated on the break in biosecurity. We suggested, that the presence of anti-CIAV antibodies may resulted from an early infection, from which chickens recovered spontaneously. This infection could weakened birds’ immune system.

The fight against *H.meleagridis* infections is based mainly on prevention and non-specific prophylactic methods (e.g., quarantine, hygienic practices, disinfection of litter and soil) (McDougald 2005). Authors suggested that the introduced treatment, and improvement of sanitary conditions might have contributed to gradual limitation of losses and control of histomonosis in the examined flock. The divergences in the epidemiology of *H.meleagridis* in chickens and turkeys (Hu et al. 2006) underline the importance of *H.gallinarum* as a vector for *H.meleagridis* in chickens. Chickens can usually become infected through the ingestion of *Histomonas*-infected eggs of caecal worm *Heterakis gallinarum* (intermediate host) or the earthworm (McDougald 2005; Hu et al. 2006)*.* In contrast to the turkeys, direct transmission (via“cloacal drinking”) has not been showed (Hu et al. 2006) or it may occur to a lesser extent than observed in turkeys (Hess et al. 2006). Therefore, it is still recommended to perform deworming (especially against *Heterakis gallinarum*) and rear chickens and turkeys separately. In general, management practices were found as very important in preventing outbreaks, but not always sufficient.

## Conclusions

All findings described in this case strongly support the interpretation that chickens have become important host for *Histomonas meleagridis.* Previously it was thought that chickens constitute mainly a reservoir and source of infection for other birds. The present study, together with the recognized lack of effective therapeutics available on the market, and limitations in prophylactics indicate that histomonosis is a re-emerging poultry disease which can pose serious health threat, decrease animal welfare and considerable economic losses in poultry production. The histomonosis will remain a formidable challenge in the years ahead.
